# Mobile Learning in Higher Education: Structural Equation Model for Good Teaching Practices

**DOI:** 10.1109/ACCESS.2020.2994967

**Published:** 2020-05-15

**Authors:** José-María Romero-Rodríguez, Inmaculada Aznar-Díaz, Francisco-Javier Hinojo-Lucena, Gerardo Gómez-García

**Affiliations:** Department of Didactics and School OrganizationUniversity of Granada 16741 18071GranadaSpain

**Keywords:** Good teaching practices, higher education, mobile devices, mobile learning, structural equation model

## Abstract

Mobile learning is a methodology that involves the use of mobile devices to carry out the teaching-learning process. In exceptional situations such as that experienced during the COVID-19 pandemic in Spain, virtual training methods take on great importance, being the main route for the education of students. The purposes of this paper were to analyse the degree of implementation of the mobile learning methodology in Spanish universities and to check the sociodemographic factors that influence the development of good teaching practices in mobile learning. Ten hypothetical relationships were established and contrasted using a structural equation model. The sample was made up of 1544 university professors from 59 Spanish universities who were asked to complete a questionnaire designed to evaluate mobile learning practices. The results indicated that the degree of implementation of mobile devices was almost 73% of the population surveyed. While the sociodemographic factors that significantly influenced the development of good teaching practices were: teacher status; type of institution; educational technology research; implementing pedagogical innovations on a regular basis; agree that mobile devices are appropriate; belief in the expansion of mobile learning. Finally, the main findings and practical implications derived from the data obtained were discussed.

## I. Introduction

Education is in a time of change, where the way of teaching and learning must be adapted to the demands of society. This implies the use of active teaching methodologies and the introduction of Information and Communication Technologies (ICT) in the classroom [1]. In specific situations, such as that experienced during the COVID-19 pandemic, online training and mobile devices become very important for carrying out the teaching-learning process. Specifically, the COVID-19 appeared in the city of Wuhan (China) in December 2019, generating an alarm worldwide with its expansion during the year 2020. The actions taken by governments have mainly been to confine the population to their homes to prevent the spread of the virus. This has had a direct impact on people’s lives and has caused teaching to move to a virtual format.

Therefore, knowing how to properly apply mobile devices to the educational context should be a requirement in times of uncertainty and social isolation as we live in Spain because of the COVID-19 pandemic. Specifically, mobile learning is defined as learning that occurs through the mediation of mobile devices, which allows a greater scope of teaching [2]. At the higher education stage, mobile learning has been linked to various benefits for students, such as increased academic performance [3]–[6] and motivation [7]–[9]. While facilitating the development of skills such as self-regulation of learning [10], [11] and cooperative work [12].

In Spanish universities the implementation of technology has had an incessant pace, where there are several experiences that involve the use of mobile devices through technologies such as augmented reality [13]–[16] or virtual reality [17]–[19]. In all these experiences the high interest of the students is highlighted and an improvement in learning is noted, since the mixed reality allows for experiential learning without having to leave the classroom or the place where the student is physically located.

However, it is still a pending subject [20], where some teachers are reluctant to implement mobile devices, derived among other motivations by the lack of training [21]–[23]. This aspect is paramount in health emergency situations where the use of technology is not optional, but it is mandatory to maintain student learning with the use of electronic media. Therefore, teacher training in technology is a priority task for universities, which should be compulsory.

On the other hand, smartphone addiction is a current problem that mainly affects university students [24]–[27]. Therefore, the implementation of mobile learning must involve the development of good teaching practice. This allows to mitigate the negative effect of the high consumption of hours dedicated to mobile devices for leisure purposes in order to redirect them to an academic and controlled use. At the same time, good teaching practices with ICT are experiences that stand out for favouring greater involvement, motivation and development of skills, being a practice that can be transferred to other contexts due to its excellence [28].

In this way, previous studies on mobile learning in higher education, focused on Spain, have been mainly dedicated to the analysis of the perceptions of teachers and students and the conditions for its adoption [29]–[36]. This differs from the knowledge about the real application of mobile devices in the Spanish university context.

Therefore, taking into consideration the current context of academic uncertainty by COVID-19 and the relevance of the use of technology to carry out university training, it is important to contextualize the results of this research at this time, which addressed the following objectives:

• To analyze the degree of implementation of the mobile learning methodology in the Spanish University
• To check the socio-demographic factors that influence the development of good teaching practices in mobile learning.

## II. Hypotheses and Research Model

**FIGURE 1.**
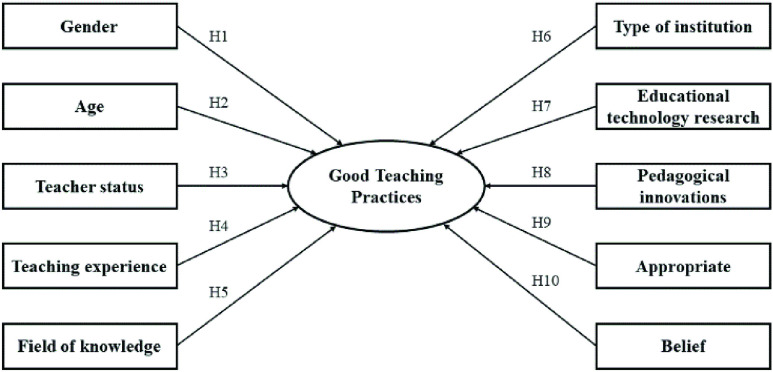
The hypothesized research model.

The extensive scientific literature on mobile learning has brought together various sociodemographic factors that influence the application of mobile devices in the classroom by teachers. Based on the premises established in previous empirical studies, the different hypotheses of the study were generated, which are included in the hypothetical research model (Figure 1). In particular, due to research linking the association of gender with the application of mobile devices [32], [37], [38], it was hypothesized: Gender is a factor that has a significant effect on good teaching practices in mobile learning (H1). Other works highlight the importance of age for the implementation of mobile devices [39]–[41], so it was of interest to establish three time-related hypotheses: Age is a factor that has a significant effect on good teaching practices in mobile learning (H2); Teacher status is a factor that has a significant effect on good teaching practices in mobile learning (H3); Teaching experience is a factor that has a significant effect on good teaching practices in mobile learning (H4).

On the other hand, specialization in a field of knowledge is also a variable to take into account in the integration of mobile devices [42], which led to the hypothesis: Field of knowledge is a factor that has a significant effect on good teaching practices in mobile learning (H5). Institutional support is fundamental for the integration of mobile devices [43], [44], where the type of institution (public or private) has been an influential factor in its application [45], being of relevance the establishment of the hypothesis: Type of institution is a factor that has a significant effect on good teaching practices in mobile learning (H6). Research and development of educational innovations in the classroom are other factors that affect the integration of mobile devices in the classroom [46]–[50]. So it was pertinent to establish the hypotheses: Educational technology research is a factor that has a significant effect on good teaching practices in mobile learning (H7); Implementing pedagogical innovations on a regular basis is a factor that has a significant effect on good teaching practices in mobile learning (H8).

Finally, the personal belief and attitude for the implementation of mobile devices is closely linked to their application [5], [8], [51]–[53], which led to the establishment of two hypotheses: Agree that mobile devices are appropriate is a factor that has a significant effect on good teaching practices in mobile learning (H9); Believing in the expansion of mobile learning is a factor that has a significant effect on good teaching practices in mobile learning (H10).

## III. Method

### A. Participants and Procedure

The design of the study was transversal, based on the application of a self-administered survey distributed via e-mail to the population of university professors who teach in the Faculties of Education of Spanish public and private universities with face-to-face teaching ($N=9655$). Prior to answering the scale, participants were informed of the purpose of the research and the anonymous processing of their data. Finally, the participating sample gave its informed consent (n = 1544). The research was conducted based on a convenience sampling design, due to the particularity of the data collection, where the scale was distributed to the whole population and everyone was free to decide their participation. The data collection period was set from May to September 2019.

A total of 1544 university professors from 59 Spanish universities participated. Of the total, 1125 professors implemented the mobile devices (72.86%) and 419 did not apply the mobile devices (27.14%). Among the reasons for non-application of mobile devices were: ignorance (45.59%); distraction (28.16%); change resistance (14.56%); uselessness (11.69%). Thus, the sample of teachers who applied the mobile devices consisted of 434 men and 691 women, between the ages of 20 and 77 (M = 4 4.66; SD = 10.36). Table 1 shows the frequency and percentage of the remaining sociodemographic data.

**TABLE 1:** Sociodemographic Data

	$n$	%
Gender		
Male	434	28.10
Female	691	44.75
Age		
20–29	79	5.11
30–39	293	18.97
40–49	374	24.22
50–59	281	18.19
60 or more	98	9.34
Teacher status		
Professor	28	1.81
Professor of University School	6	0.38
Senior Lecturer	215	13.92
Senior Lecturer of University School	10	0.64
Lecturer	211	13.66
Assistant Professor PhD	149	9.65
Assistant Professor	13	0.84
Interim Substitute Professor	64	4.14
Associate Lecturer	306	19.81
Adjunct Professor	21	1.36
Postdoctoral	6	0.38
Pre-doctoral	63	4.08
Visiting Professor	5	0.32
Collaborating Professor	26	1.68
Emeritus Professor	2	0.12
Teaching experience		
1–5	291	18.84
6–10	241	15.60
11–15	154	9.97
16–20	133	8.61
21–25	114	7.38
26 or more	192	12.43
Field of knowledge		
Didactics of Body Expression	66	4.27
Didactics of Musical Expression	42	2.72
Didactics of Plastic Expression	41	2.65
Didactics of Language and Literature	110	7.12
Didactics of Experimental Sciences	86	5.56
Didactics of Social Sciences	72	4.66
Didactics of Mathematics	59	3.82
Didactics and School Organization	241	15.60
Physical and Sport Education	78	5.05
Research and Diagnostic Methods in Education	81	5.24
Evolutionary and Educational Psychology	153	9.90
Theory and History of Education	96	6.21
Type of institution		
Public	917	59.39
Private	208	13.47
Educational technology research		
Yes	419	27.13
No	706	45.72
Implementing pedagogical innovations on a regular basis		
Yes	1063	68.84
No	62	4.01
Mobile devices are appropriate		
Yes	1052	68.13
No	73	4.72
Mobile learning expansion (belief)		
Yes	905	58.61
No	220	14.24

### B. Measure

An ad hoc questionnaire was used for data collection, as there was no instrument to evaluate good teaching practices in mobile learning [35]. The scale created, called Analysis of M-learning practices at the University (APMU), evaluated the mobile learning practices implemented by university teachers by establishing and refining quality indicators to evaluate good teaching practices in mobile learning [54].

The 16 items on the scale were grouped into five dimensions: mobile devices (1-3); digital competence (4-5); knowledge construction (6-9); cooperative work (10-12); good use of technology (13-16). The response mode was by means of a four-level Likert scale ($1=$ never; $4=$ always). So the scale scores ranged from 16 to 64 points, where a higher score meant that the implementation of mobile devices in the classroom led to good teaching practice. These items are:

1. Do students have a mobile device to work in the classroom (smartphone, tablet or laptop)?
2. Do students use mobile devices in the classroom during subject time, i.e. do they use them in the tasks that require their use?
3. Do you make a didactic use of the mobile device in the activities you develop in the classroom, that is, do you take into account the functionalities of the mobile device in the teaching and learning process?
4. Do the activities planned with mobile devices allow students to produce digital content?
5. Do the activities planned with mobile devices allow students to share information socially?
6. In the activities that you implement through mobile devices, do you consider that there is a greater understanding of the content by students?
7. Do the activities you implement through mobile devices allow you to track the student’s learning process?
8. Does it provide feedback to students in the different activities that take place with mobile devices?
9. Do the activities, tasks or projects developed through the mobile device encourage the student to reflect on his/her own learning?
10. Do the activities developed through mobile devices encourage cooperative work?
11. Do the activities planned with mobile devices encourage interaction between students?
12. Do the activities proposed with mobile devices allow for group decision-making?
13. When doing any activity that requires the use of the mobile device, do you warn students about the risks of improper use?
14. Do you teach students to use available filters so that mobile devices do not display adult content?
15. When you apply a methodology based on mobile learning, do you establish prevention guidelines to avoid addictive behaviours to mobile devices?
16. Does it inform students about the health consequences for children of inappropriate use of a mobile device at an early age?

To estimate the psychometric properties and internal consistency of the instrument, convergent and discriminant validity and reliability were calculated using Cronbach’s Alpha coefficient (Tabla 2 y Tabla 3). Convergent and discriminant validity were assessed using the measurement model [55]. Adequate factor loads were obtained, so convergent and discriminant validity of constructs was verified [56].

The Kaiser Meyer Olkin (KMO) measure of sampling adequacy was also adequate (KMO = 0.844). Bartlett’s sphericity test obtained the values of $\chi ^{2}=6194.333$; df = 120; $p=0.000$.

### C. Data Analysis

We calculated the mean and standard deviation for each socio-demographic factor and checked whether there were statistically significant differences between the groups. For this purpose, the T test was used for independent samples when it was a comparison between two groups and the ANOVA test when there were more than two groups.

Hypothesis testing was performed using path analysis. In it, the different relationships with the good teaching practices in mobile learning were established and it was checked if each relationship was significant. However, before the establishment of the structural equation model (SEM), the hypothesis of multivariate normality was confirmed as a precondition through Mardia’s coefficient [57].

Also, different goodness-of-fit indices were collected to confirm the adequacy of the SEM [58]: Chi-square ($\chi ^{2}$); degrees of freedom (*df*); the ratio $\chi ^{2}$/*df*; goodness-of-fit index (GFI); root mean squared error of approximation (RMSEA); normalised fit index (NFI); comparative fit index (CFI); adjusted goodness-of-fit index (AGFI).

The various analyses were performed with Microsoft Excel Professional Plus 2013 (Microsoft, Redmond, WA) and the statistical packages IBM SPSS and IBM SPSS Amos, version 24 (IBM Corp., Armonk, NY).

## IV. Results

Data concerning the validity and reliability of the instrument showed adequate psychometric properties on the APMU scale. In convergent validity, average variance extracted obtained an adequate value for all items (AVE > 0.5) [59]. While the composite reliability values of the items were also adequate, where values above or close to the appropriate (CR > 0.8) (Table 2). On the other hand, the reliability of the scale calculated by Cronbach’s Alpha coefficient was at correct values ($\alpha =0.834$).

**TABLE 2:** Convergent Validity Measures and Reliability

Construct	Item	Factor Loading	CR	AVE	$\alpha $	Total $\alpha $
MD	MD1	0.878	0.830	0.623	0.655	0.834
	MD2	0.821				
	MD3	0.652				
DC	DC1	0.767	0.795	0.661	0.605	
	DC2	0.857				
KC	KC1	0.667	0.830	0.553	0.742	
	KC2	0.856				
	KC3	0.749				
	KC4	0.690				
CW	CW1	0.820	0.888	0.726	0.830	
	CW2	0.877				
	CW3	0.859				
GUT	GUT1	0.765	0.898	0.689	0.843	
	GUT2	0.846				
	GUT3	0.882				
	GUT4	0.823				

Note: MD=mobile devices; DC = digital competence; KC = knowledge construction; CW = cooperative work; GUT = good use of technology.

For the discriminant validity analysis, the square root of AVE was taken to correlate the latent constructs (Table 3). The discrimination of each factor was verified, which represented a different dimension. This led to the psychometric characteristics of the instrument being acceptable [60].

**TABLE 3:** Discriminant Validity Measures

	MD	DC	KC	CW	GUT
MD	0.789				
DC	0.448	0.813			
KC	0.650	0.579	0.744		
CW	0.435	0.433	0.570	0.852	
GUT	0.187	0.345	0.303	0.227	0.830

Note: Diagonals represent the average variance extracted, while the other matrix entries represent the squared correlations.

In relation to the averages obtained for each socio-demographic factor, the significant differences were field of knowledge ($p=0.000$), educational technology research ($p=0.000$), implementing pedagogical innovations on a regular basis ($p=0.000$), mobile devices are appropriate ($p=0.000$), and belief in the expansion of mobile learning ($p=0.000$). With respect to the highest average scores by factor, these were collected from women (M = 45.07), age 50–59 (M = 45.43), Collaborating Professor (M = 45.96), teaching experience of 21–25 years (M = 46.13), Department of Didactics of Plastic Expression (M = 47.88), private universities (M = 45.77), the research line is educational technology research (M = 48.46), implementing pedagogical innovations on a regular basis (M = 45.40), agree that mobile devices are appropriate (M = 4 5.33), and belief in the expansion of mobile learning (M = 45.75) (Table 4).

**TABLE 4:** Descriptive Statistical Data and Differences Between Groups

	M	SD	$p$
Gender			
Male	45.06	7.903	0.981
Female	45.07	7.391	
Age			
20–29	45.18	7.082	0.784
30–39	44.91	7.275	
40–49	45.09	7.822	
50–59	45.43	7.582	
60 or more	44.32	8.094	
Teacher status			
Professor	44.36	8.719	0.901
Professor of University School	43.17	9.806	
Senior Lecturer	44.80	8.030	
Senior Lecturer of University School	34.40	8.462	
Lecturer	45.11	7.276	
Assistant Professor PhD	44.52	7.590	
Assistant Professor	45.08	8.129	
Interim Substitute Professor	44.89	8.218	
Associate Lecturer	45.62	7.356	
Adjunct Professor	42.48	9.152	
Postdoctoral	45	6.419	
Pre-doctoral	45.89	6.802	
Visiting Professor	46	5.788	
Collaborating Professor	45.96	5.596	
Emeritus Professor	42	21.213	
Teaching experience			
1–5	44.43	7.608	0.269
6–10	45.03	7.136	
11–15	45.81	6.906	
16–20	45.32	7.731	
21–25	46.13	8.438	
26 or more	44.69	7.968	
Field of knowledge			
Didactics of Body Expression	43.02	7.128	0.000
Didactics of Musical Expression	45.90	7.867	
Didactics of Plastic Expression	47.88	5.997	
Didactics of Language and Literature	46.83	6.618	
Didactics of Experimental Sciences	44.91	8.125	
Didactics of Social Sciences	43.67	6.751	
Didactics of Mathematics	42.17	6.934	
Didactics and School Organization	47.20	7.602	
Physical and Sport Education	43.78	8.449	
Research and Diagnostic Methods in Education	45.44	6.841	
Evolutionary and Educational Psychology	43.39	7.340	
Theory and History of Education	43.91	8.171	
Type of institution			
Public	44.91	7.629	0.137
Private	45.77	7.387	
Educational technology research			
Yes	48.46	6.764	0.000
No	43.05	7.333	
Implementing pedagogical innovations on a regular basis			
Yes	45.40	7.470	0.000
No	39.32	7.366	
Mobile devices are appropriate			
Yes	45.33	7.354	0.000
No	41.33	9.720	
Mobile learning expansion (belief)			
Yes	45.75	7.413	0.000
No	42.27	7.678	

Note: $p$ calculated through the T and ANOVA test.

The establishment of the SEM implied, on the one hand, the confirmation of the hypothesis of multivariate normality of the data (Mardia = 25,697). This coefficient was less than 288, extracted from p $^\ast $(p + 2), with “p” being the number of total variables in the scale (16) [61]. And on the other hand, the adequacy of the goodness-of-fit indexes: $\chi ^{2}=1.691$; *df* = 2; the ratio $\chi ^{2}$/*df* was 0.8455; GFI = 1; RMSEA = 0.000; NFI = 0.999; CFI = 1; AGFI = 0.991.

Finally, a total of six hypotheses of the 10 hypothetical relationships were supported (Table 5). Therefore, the relationship established between the following factors and good teaching practices in mobile learning was significant: Teacher status (H3); Type of institution (H6); educational technology research (H7); implementing pedagogical innovations on a regular basis (H8); agree that mobile devices are appropriate (H9); belief in the expansion of mobile learning (H10). The hypotheses that were not supported were rejected.

**TABLE 5:** Hypothesis Testing Results

	Relationship	Path coeff.	CR	$p$	Results
H1	GTP $\leftarrow $ Gender	0.020	0.742	0.458	Rejected
H2	GTP $\leftarrow $ Age	−0.050	−1.250	0.211	Rejected
H3	GTP $\leftarrow $ Teacher status	0.067	2.083	0.037	Supported
H4	GTP $\leftarrow $ Teaching experience	0.076	1.787	0.074	Rejected
H5	GTP $\leftarrow $ Field of knowledge	−0.030	−1.103	0.270	Rejected
H6	GTP $\leftarrow $ Type of institution	0.054	1.981	0.048	Supported
H7	GTP $\leftarrow $ Edu. Tech. research	−0.324	−11.78	***	Supported
H8	GTP $\leftarrow $ Pedagogical innovations	−0.116	−4.206	***	Supported
H9	GTP $\leftarrow $ Appropriate	−0.096	−3.503	***	Supported
H10	GTP $\leftarrow $ Belief	−0.154	−5.615	***	Supported

Note: GTP=good teaching practices; CR=critical radio; ***Significant at $p < 0.001$.

**FIGURE 2.**
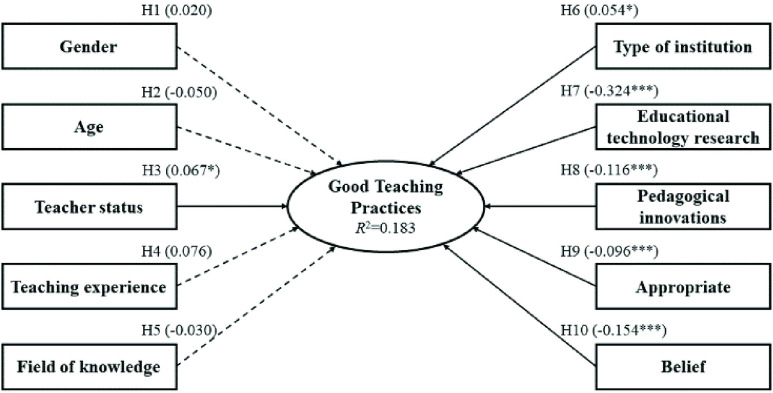
Structural equation model. Note: $^\ast $Significant at $p < 0.05$; $^{\ast \ast \ast }$ Significant at $p < 0.001$.

The SEM graphically exemplified the relationship between the dependent variables that were significant for good teaching practices in mobile learning (Figure 2). The coefficient of determination ($R^{2}$) of the model was 0.183, with a percentage of variation of 18.3%. Non-significant relationships were shown with broken lines.

## V. Discussion

The data showed a rather optimistic picture regarding the implementation of mobile devices in Spanish university education, since their application was of almost 73% of the total sample. However, taking into consideration some of the reasons expressed by teachers who do not apply mobile devices, it is clear that there is still a long way to go for their generalization [20]. Among these apparent reasons, ignorance stands out as the main premise of the lack of teacher training [21]–[23]. It is therefore essential to encourage teacher training in technological matters at universities, and even more so in the current context.

Distraction, change resistance and uselessness were other reasons highlighted. As for the belief that they distract students, this can be mitigated if introduced in an appropriate and controlled manner by establishing a schedule for their use. The biggest problem is the resistance to change presented by some teachers, where they perceive technology as something useless and have no intention of changing their teaching methodology.

In terms of the instrument used, the APMU scale was positioned as a valid and reliable tool for assessing mobile learning practices and detecting the development of good teaching practices. Its psychometric properties make this scale possible as a measure that can be used in future studies on good teaching practices in mobile learning.

In particular, given the contrast of hypotheses, most of them were accepted (six of 10 hypotheses); see Table 5 and Figure 2. As for gender (H1), it did not have any influence on the development of good practice despite what was highlighted in previous studies [32], [37], [38]. However, the women obtained a slightly higher average, but it was not significant. This indicated that gender does not predetermine good teaching practices.

As for the hypotheses concerning time (H2, H3, H4), only H3 was accepted. Thus, age (H2) had no influence on the development of good teaching practices, being developed by teachers of any age. This contrasted with data from previous studies that related age to the application of mobile devices [39]–[41]. Teaching experience (H4) was also not a factor. This reinforced the premise that age does not influence. However, teacher status (H3) had a significant relationship to the development of good teaching practices. So being in one category or another influences the excellent application of mobile devices. In particular, the Collaborating Teachers obtained the highest average. This category is characterized by being a person external to the institution who regularly participates in teaching tasks.

The field of knowledge hypothesis (H5) was not supported. However, if significant differences were found between the different areas, highlighting the Department of Didactics of Plastic Expression. The particular case of this area of knowledge can perhaps be explained by the profile of these teachers, who often require the application of technological resources in the classroom due to the type of subjects they teach. However, a priori the field of knowledge was not associated as an influential factor, in contrast to the data that affirmed this premise [42].

On the other hand, the hypothesis regarding the type of institution (H6) was supported, stressing that belonging to a private university influences the development of good teaching practices [45]. This may be because institutional and resource support from private universities could be greater, and this facilitates the application of technological resources in the classroom. The data from this study support this premise, since the average obtained by these teachers was higher. In future studies it would be interesting to bring together these differences between public and private universities.

The two hypotheses linked to research and teaching innovation were supported (H7, H8). The fact that teachers’ line of research is educational technology is a differentiating factor in the development of good teaching practices. This can be influenced by the knowledge that teachers possess in this area, which plays in their favor in the teaching work they develop. Furthermore, the implementation of pedagogical innovations on a regular basis is another indicator that significantly influences the development of good teaching practices. This is due to the combination of mobile learning with other active methodologies [46]–[50]. Thus, methodological complementarity affects the improvement of student learning, who experience different methods that favour the development of skills.

Finally, hypotheses related to belief and personal attitude towards mobile devices and mobile learning were supported (H9, H10). Therefore, the perceived usefulness to this resource is one of the main factors for its adoption [5], [8], [51]–[53]. In addition, being aware of the advantages of using mobile devices and the belief in their current and future relevance were two of the factors that influenced the development of good teaching practices in mobile learning.

### A. Limitations and Prospective

The limitations of this study are grouped around two main limitations: the transversality of the data and the limited sample size in some sociodemographic factors. As for the first, the data reflects a static picture of the Spanish University regarding the implementation of mobile devices at a particular time. Therefore, no conclusions and inferences of temporality can be drawn. For this, it would be convenient to replicate the study over time, with a longitudinal. This will be proven over time, when the study is replicated over an extended period of years.

As for the limited sample size, in some strata populations there is a sample decompensation with respect to others. However, it was decided to maintain these cases to ensure the representativeness of all sectors and not to exclude any. This allowed the data to be as true to reality as possible. It was therefore important to reflect these cases in order to support the research with solid and reliable data on good teaching practices in mobile learning at the University.

For its part, this study opens the doors and establishes the beginnings of other derived research that can be focused on: (i) the evaluation of good teaching practices in mobile learning; (ii) replication of this study in other contexts to compare the results obtained; (iii) the identification of concrete experiences of good teaching practices; (iv) the application of the influencing factors in this study to the development of training plans for teachers.

## VI. Conclusions

The use of mobile devices to mediate learning becomes very relevant in virtual training. Situations such as that arising from COVID-19 highlight the importance of training teachers in technological skills and the proper use of technology. Faced with this panorama of uncertainty, mobile learning is a useful methodology to develop learning in an active way and with total normality in exceptional situations.

In this paper we responded to the objectives set, we analyzed the degree of implementation of the mobile learning methodology in the Spanish University, where we obtained the percentage of implementation and the main reasons why teachers do not use mobile devices. In turn, the sociodemographic factors that influenced the development of good teaching practices in mobile learning were verified, highlighting six: teacher status; type of institution; educational technology research; implementing pedagogical innovations on a regular basis; agree that mobile devices are appropriate; belief in the expansion of mobile learning.

Finally, the current educational landscape and vision of education must go hand in hand with the adoption of technology. Nevertheless, this adoption cannot be limited to the simple introduction, but must be accompanied by the development of good teaching practices, which will serve as a reference for teachers who want to start or improve their teaching activity with ICT.
